# Slow advancement of the endotracheal tube during fiberoptic-guided tracheal intubation reduces the severity of postoperative sore throat

**DOI:** 10.1038/s41598-023-34879-1

**Published:** 2023-05-12

**Authors:** Hyunjee Kim, Jeong Eon Kim, Yeongun Kim, Seong Wook Hong, Hoon Jung

**Affiliations:** Department of Anesthesiology and Pain Medicine, School of Medicine, Kyungpook National University, 130 Dongdeok-Ro, Jung-Gu, Daegu, 41944 Republic of Korea

**Keywords:** Medical research, Risk factors, Signs and symptoms

## Abstract

Although fiberoptic-guided tracheal intubation is a gentler method than using a direct laryngoscope, injury may occur owing to impingement between the distal edge of the endotracheal tube and the glottis. This study aimed to investigate the effects of endotracheal tube advancement speed during fiberoptic-guided intubation on airway symptoms postoperatively. We randomized patients scheduled for laparoscopic gynecological surgery to either Group C or S. When advancing the tube over the bronchoscope, the operator advanced the tube at a normal speed in Group C and at a slower speed in Group S. The speed in Group S was approximately half that in Group C. The target outcomes were the severity of postoperative sore throat, hoarseness, and cough. Patients in Group C experienced a more severe sore throat than those in Group S at 3 and 24 h postoperatively (*p* = 0.001 and *p* = 0.012, respectively). However, the severity of hoarseness and cough postoperatively were not significantly different between the groups. In conclusion, the slow advancement of the endotracheal tube during fiberoptic-guided intubation can reduce sore throat severity.

## Introduction

Postoperative sore throat is a common adverse effect of general anesthesia that is characterized by several signs and symptoms, including laryngitis, tracheitis, hoarseness, and cough. The mucosal injury location varies depending on the device used for airway management, with the type of endotracheal tube and the approach to airway management being the most significant physical factors^1^.

When performing tracheal intubation using a direct laryngoscope, direct stimulation through the blade may damage the airway tissue. In contrast, when performing tracheal intubation under fiberoptic bronchoscope guidance, injury may occur because the tube’s outer diameter is larger than that of the bronchoscope, thereby causing impingement against the glottis^2,3^. In a study comparing fiberoptic-guided tracheal intubation with direct laryngoscopy with respect to postoperative sore throat, the authors suggested that the blind passage of the endotracheal tube through the glottis during fiberoptic-guided intubation resulted in sore throat^4^.

This study aimed to determine a method for reducing the severity of postoperative sore throat following endotracheal intubation under fiberoptic bronchoscope guidance. To reduce impingement during fiberoptic-guided tracheal intubation, several methods have been suggested^2,3^; however, the relationship between tracheal tube insertion speed and postoperative sore throat remains unclear. We hypothesized that slower tube tip insertion through the glottis would cause less trauma to the tissue, thereby compensating for fixed factors, including the blind passage and the gap between the tube and the bronchoscope.

## Methods

### Study design

This study was approved by the Institutional Review Board of Kyungpook National University Hospital (KNUH 2020-11-069; February 8, 2021) and was registered at cris.nih.go.kr (KCT0006673; first registration on 18/10/2021) prior to enrollment. This study was designed as a prospective, randomized, controlled trial and was conducted at Kyungpook National University Hospital in accordance with the Declaration of Helsinki. Written informed consent was obtained preoperatively from each participant. This study adheres to the Consolidated Standards of Reporting Trials guidelines.

### Inclusion and exclusion criteria

The following were the inclusion criteria: patients who were undergoing laparoscopic gynecological surgery under general anesthesia, aged 18–65 years, height of 155–167 cm, nonsmokers, expected anesthesia duration of ≤ 3 h, and an American Society of Anesthesiologists physical status of class I–II. The following were the exclusion criteria: patients with severe obesity (body mass index ≥ 30 kg/m^2^), thyromental distance of < 6.5 cm, Mallampati class III or IV, history of recent upper respiratory infection or sore throat within 7 days preoperatively, use of steroid medications, and pregnancy. Furthermore, patients with an anesthesia duration of < 1 or > 3 h, had airway spasms or bucking episodes during surgery and extubation, had discontinuation of intravenous patient-controlled analgesia (IV-PCA), or used analgesics other than IV-PCA were excluded from the analyses.

### Study procedures

Patients were categorized into the following two groups: Group C (control group) and Group S (slow endotracheal tube advancement). A predetermined computer-generated random number sequence was used to generate a randomization sequence for the two groups in a ratio of 1:1. Group allocation was performed on the morning of the day of surgery using sealed, number-coded envelopes.

On arrival at the operating room, electrocardiogram, sphygmomanometer, pulse oximeter, bispectral index (BIS), and temperature were monitored. All patients were preoxygenated with 100% oxygen for 3 min. General anesthesia was induced with intravenous propofol 1.5–2 mg/kg, a target-controlled intravenous infusion of remifentanil 3.5–4.5 ng/mL, and intravenous rocuronium 0.6 mg/kg. After reducing the BIS to < 50 and ablation of the train-of-four (TOF) response (TOF 0), an investigator performed fiberoptic‐guided tracheal intubation. A Shiley™ Lo-Contour flexible reinforced endotracheal tube (Covidien Ireland Limited, Tullamore, Ireland), which had an outer diameter of 7.0 mm, was used for tracheal intubation. The endotracheal tube was premounted near the proximal site of the bronchoscope (Olympus LF-GP, Olympus Optical Co., Tokyo, Japan), and the bronchoscope was inserted into the trachea through the patient’s mouth while an assistant pulled the patient’s jaw.

**Figure 1:**
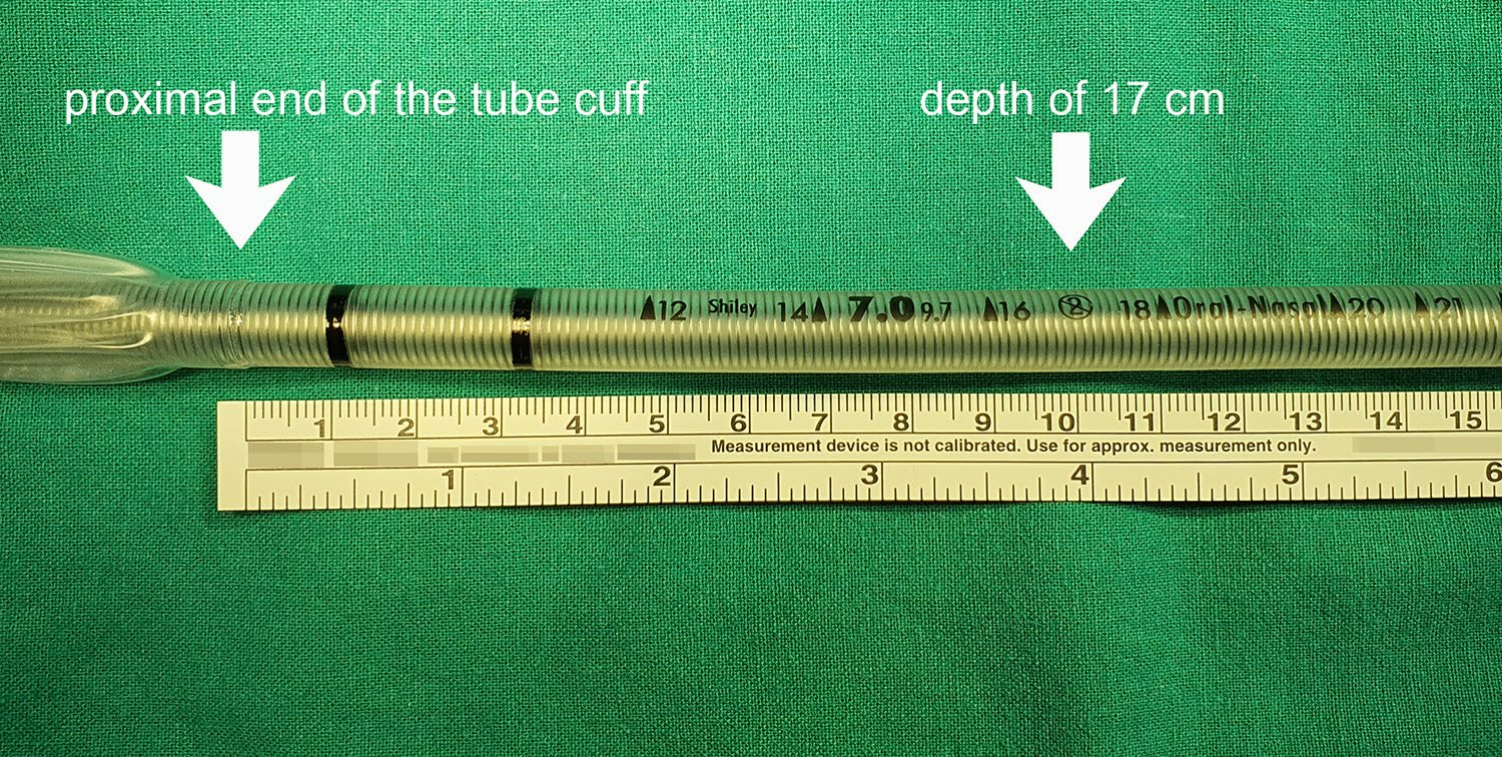
Start and end of time measurement of endotracheal tube advancement (arrows).

Once the bronchoscope was inserted into the trachea and reached the level of the carina, the endotracheal tube was advanced over the bronchoscope to a depth where the proximal end of the tube cuff was in contact with the patient’s incisors. The tube was subsequently advanced by an additional 10 cm to a depth of 17 cm from the patient’s incisors; the time required to perform this step was measured^5,6^ (Fig. 1). In Group C, the operator advanced the endotracheal tube at a normal speed, whereas in Group S, the tube was advanced at a much slower speed. The final depth of the endotracheal tube was adjusted by bronchoscopic examination.

Medical staff who did not participate in the study performed the maintenance of general anesthesia and emergence. Sevoflurane in O_2_/air, target-controlled intravenous infusion of remifentanil (2.5–3.5 ng/mL), and intravenous rocuronium were administered to all patients. The cuff pressure of the endotracheal tube was maintained between 20 and 25 cm H_2_O using a manual cuff pressure manometer (Cuff Manometer, Mallinckrodt Medical, Athlone, Ireland). Intravenous sugammadex was administered at the end of the surgery. All patients received IV-PCA with fentanyl 600 µg and ketorolac 240 mg diluted with 100 mL of normal saline, at a 2-mL/h basal infusion rate, 1-mL on-demand bolus, and 10-min lockout interval.

### Study assessments

The primary outcome of the study was the difference in postoperative sore throat scores between the groups, and the secondary outcome was the difference in the severity of postoperative hoarseness and cough.

A designated investigator who was blinded to the group allocation evaluated postoperative sore throat, hoarseness, and cough at 3 and 24 h following surgery.

Sore throat was defined as continuous throat pain and estimated using a visual analog scale (VAS) on a 100-mm scale. Postoperative hoarseness and cough were assessed using a numerical rating scale. Postoperative hoarseness was measured as follows: 0 = no hoarseness, 1 = experienced only by the patient, 2 = evident to the evaluator, and 3 = difficult to vocalize^7,8^. Postoperative cough was measured as follows: 0 = none, 1 = mild (less than a common cold), 2 = moderate (similar to a common cold), and 3 = severe (more than a common cold)^9^. The total use of analgesics through IV-PCA or other approaches was also recorded.

### Statistical analyses

Statistical analyses were performed using Statistical Package for the Social Sciences (version 24, IBM Corp, Armonk, NY, USA). The sample size was calculated from a pilot study, wherein the severity of sore throat 3 h postoperatively (mean ± standard deviation) was 20.5 ± 11.6 and 11.6 ± 11.0 in Groups C and S, respectively (n = 6 per group). A two-tailed α value of 0.05 and a power of 80% were assumed; the required sample size was determined to be 56 (28 per group) to assess the severity of sore throat 3 h postoperatively and demonstrate a statistically significant difference between the two groups (effect size = 0.79). Thus, assuming potential dropouts, a target sample size of 33 patients per group was planned. None of the patients in the pilot study was included in the present study.

To present demographic data and target measurements, descriptive statistics were used. To compare the demographic data, time measurement, and amount of IV-PCA used, an independent Student’s *t*-test was performed according to the characteristics and distribution of the data. To assess the homogeneity of variances, Levene’s test was performed. The VAS scores of postoperative sore throat were compared using the Mann–Whitney U test. A *p* value of < 0.05 was considered statistically significant.

## Results

**Figure 2:**
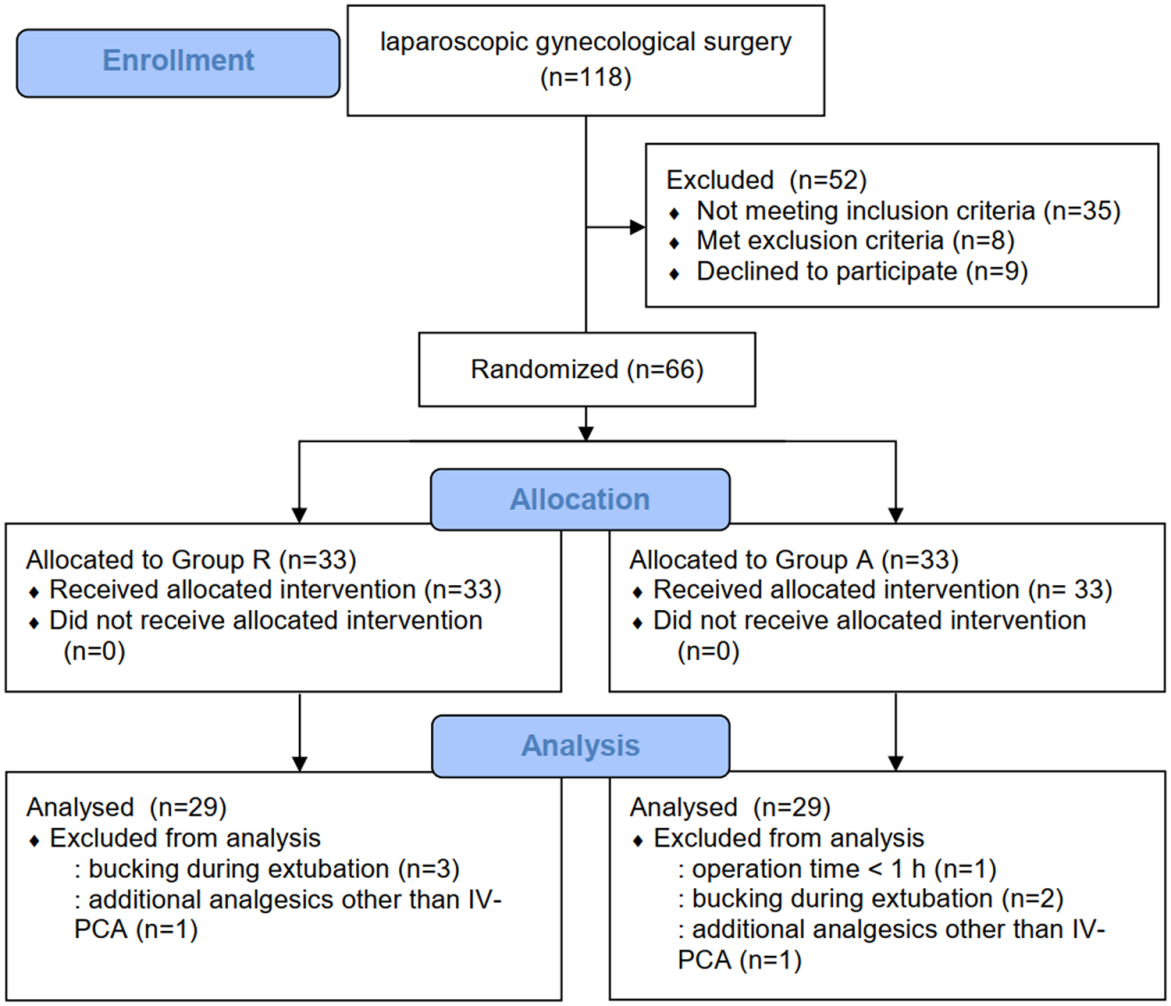
Patients’ flow diagram.

Of the 118 patients assessed for eligibility, 43 were excluded on the basis of the exclusion criteria, and 9 refused to participate. Therefore, a total of 66 patients were finally enrolled in the study. One patient was excluded because her anesthesia lasted < 1 h, five patients were excluded owing to bucking episodes before extubation, and two patients were excluded because of requiring additional analgesics other than IV-PCA postoperatively. Therefore, 29 patients per group were included in the final analysis (Fig. 2).

The descriptive statistics of the patients’ demographic data, time required for endotracheal tube insertion (reflecting the insertion speed), anesthesia duration, and total use of analgesics through IV-PCA until 24 h are shown in Table 1. No significant differences were observed in the demographic characteristics between the groups. Moreover, the anesthesia duration and IV-PCA use were similar between the groups. The time required for the endotracheal tube to advance through the designated section, the main intervention factor in this study, were 9.9 ± 1.6 and 18.7 ± 1.2 s in Groups C and S, respectively, indicating that the tube insertion speed in Group S was approximately half of that in Group C.

**Table 1:** Demographic data and other parameters (mean ± SD).

	Group C (n = 29)	Group S (n = 29)	*P* value
Age (years)	48 ± 11.3	50 ± 9.9	0.58
Height (cm)	161 ± 3.5	160 ± 3.8	0.78
Weight (kg)	58 ± 6.9	58 ± 7.1	0.91
Insertion time (s)	9.9 ± 1.6	18.7 ± 1.2	< 0.001
Anesthesia time (min)	130 ± 23.6	130 ± 22.1	0.95
IV-PCA (mL) (24 h)	54 ± 6.3	56 ± 6.2	0.53

Values are mean ± SD.

IV-PCA; intravenous patient controlled analgesia.

The patients’ sore throat, hoarseness, and cough scores at 3 and 24 h postoperatively are presented in Table 2. More patients in Group C experienced severe sore throat than those in Group S at 3 and 24 h postoperatively (*p* = 0.001 and *p* = 0.012, respectively). However, the severity of hoarseness and cough postoperatively did not significantly differ between the groups.

**Table 2:** Postoperative sore throat, hoarseness, and cough scores of the patients.

	Group C (n = 29)	Group S (n = 29)	*P* value
3 h sore throat severity (VAS, 0–100)	17.3 ± 10.5	8.6 ± 8.6	0.001
24 h sore throat severity (VAS, 0–100)	11.9 ± 9.2	6.4 ± 7.2	0.012
3 h hoarseness (NRS, 0/1/2/3)	28/1/0/0	27/2/0/0	–
24 h hoarseness (NRS, 0/1/2/3)	29/0/0/0	28/1/0/0	–
3 h cough (NRS, 0/1/2/3)	26/3/0/0	27/1/1/0	–
24 h cough (NRS, 0/1/2/3)	28/1/0/0	29/0/0/0	–

Values are mean ± SD or number of patients.

## Discussion

This study demonstrated that slow advancement of the endotracheal tube during fiberoptic-guided intubation decreases the severity of postoperative sore throat in patients undergoing gynecological surgery. The severity of postoperative sore throat was lower in the low-speed group, wherein the endotracheal tube advancement speed was twice slower than that in the control group. Since the gap between the bronchoscope and the endotracheal tube may cause the distal edge of the tube to collide with the tissue around the glottis, the slow advancement of the tube is considered to reduce tissue injury and cause less severe sore throat. Despite the difference in tube advancing speeds between the groups, no difference was noted in the severity of postoperative hoarseness and cough. This can be explained by the low incidence of hoarseness and cough in the surgery and anesthesia groups of this study.

**Figure 3:**
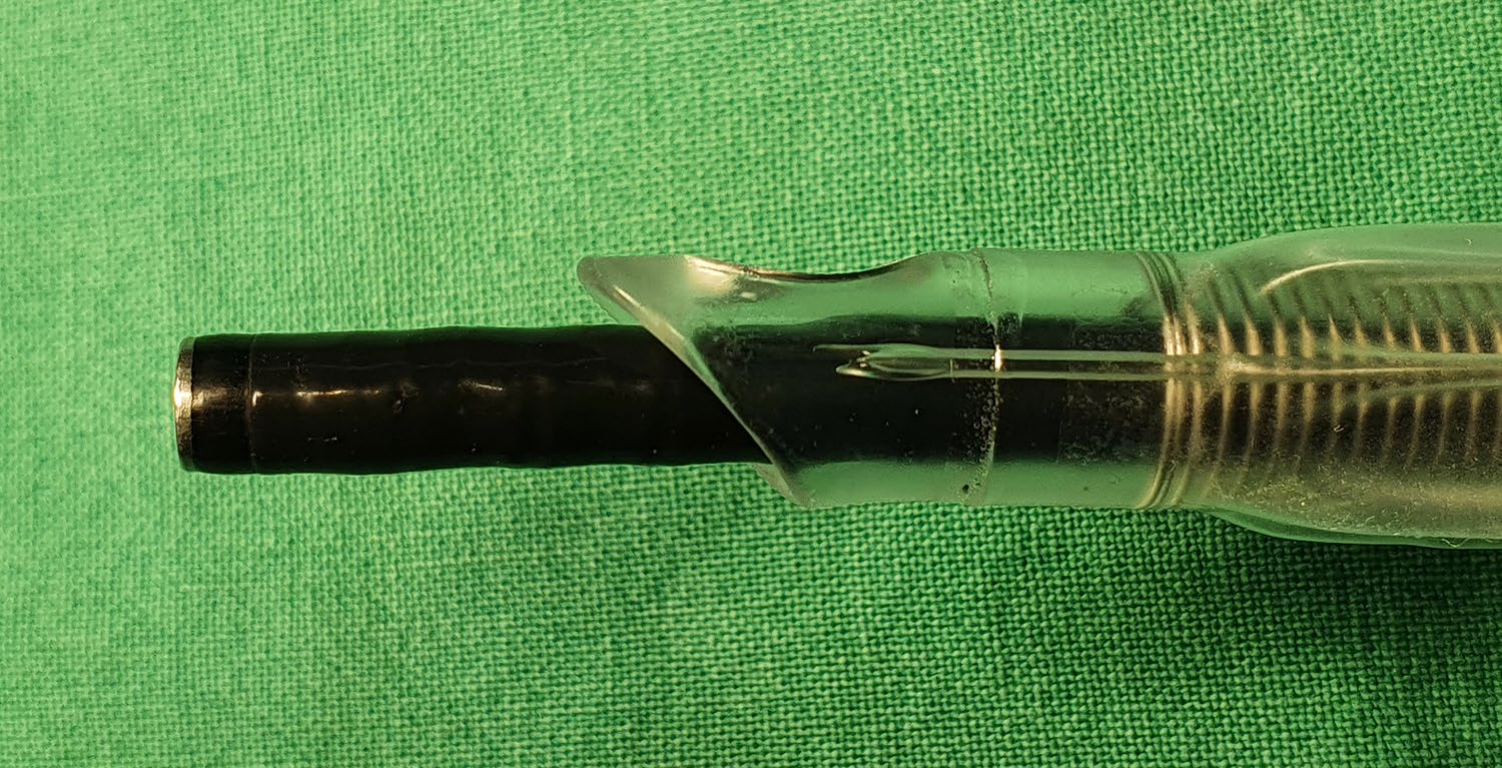
Gap between the bronchoscope and the endotracheal tube.

When the endotracheal tube is advanced over a bronchoscope during fiberoptic-guided tracheal intubation, the distal end of the tube is beyond the operator’s field of view. Moreover, the tube diameter is larger than that of the bronchoscope; this gap can cause the leading edge of the tube to contact and get caught on glottic structures^2^ (Fig. 3). The impingement of the tube on laryngeal structures may interfere with tube insertion and increase the risk of tissue injury. In terms of ease of tube advancement and decreasing laryngeal impingement, using a tracheal tube with the smallest appropriate external diameter^10^, endotracheal tube with a tapered tip^11^, or Parker Flex-Tip endotracheal tube^12^ is superior to using standard polyvinylchloride tracheal tubes. In this study, the use of a 7.0-mm endotracheal tube in all patients decreased the effects of the tube diameter on the comparative analysis of the results.

Postoperative sore throat is related to several significant physical factors. One factor includes the process of airway management, endotracheal tube, or cuff design^1^. Female sex, younger age, and gynecological surgery increase the incidence of postoperative sore throat^13^. In the present study, all participants were females undergoing laparoscopic gynecological surgery, and the distribution of the patients’ age between the groups was similar in the comparative analysis. It has been reported that the type of single-lumen tracheal tube does not affect the sore throat^14^. No significant difference in the incidence of postoperative sore throat or between the Parker Flex-Tip tube and standard endotracheal tube was observed. In the present study, we intended to control for differences in physical factors that occur in normal clinical practice other than the tube advancement speed.

The endotracheal tube advancement speed over the bronchoscope varies among clinicians, clinical situations, or assistants who may be asked to advance the tube. If the process of bronchoscope insertion into the trachea becomes challenging or time-consuming, the tube insertion may be rushed to avoid prolonged apnea time and the subsequent risk of hypoxia. When oxygen saturation is decreased during the procedure, securing the airway should be an urgent priority. However, based on the results of this study, if operators can recognize a slow procedure to reduce the severity of postoperative sore throat, then it can be implemented in general clinical practice to reduce airway-related complications and aid in postoperative recovery.

In this study, the tube insertion time showed a difference of approximately 9 s between the two groups, and the time taken from bronchoscope insertion to endotracheal intubation completion was within approximately 70 s in all participants. Considering that it took more time than the usual procedure for accurate time measurement during the study, that 100% saturation was maintained for several minutes during apnea after preoxygenation^15^, and that the partial pressure of carbon dioxide increased by 3–6 mmHg per minute during apnea^16^, slow tube advancement can be safely performed for experienced bronchoscopy operators.

This study had two limitations. First, the subjects were limited to women undergoing gynecological surgery. However, this contributed to adjustment for confounding variables, including surgery type and sex, which affect the postoperative sore throat. Furthermore, since the size of the endotracheal tube was fixed, the gap between the bronchoscope and the tube, which is a major factor that causes difficulties during fiberoptic-guided intubation, was kept constant in all the subjects. However, additional studies that are inclusive of all sexes will be helpful for the general and broad application of the results of this study. Second, the tube advancement speed made it challenging to set a specific standard. Furthermore, it is not clinically easy to keep the tube advancement speed constant during the procedure, although the advancement speed near the glottis is the most significant factor. In this study, the operator was instructed to move the tube consciously and very slowly within the experimental group, and the degree of slowness was confirmed as the average speed. To establish a basis for specific speed guidelines for clinical application, further studies are needed. Third, the occurrence of sore throat following flexible bronchoscopy for examination and procedure has been reported in approximately 10%^17^. Compared with this study, there will be differences in terms of the cause and degree of sore throat that occur when bronchoscopic procedures are performed in a conscious or sedated state. However, the soft tissue around the airway may be damaged during the bronchoscope insertion process in this study as well, and the possibility of bronchoscope insertion-induced sore throat cannot be excluded; therefore, it is considered as an unadjusted confounding factor in sore throat development.

In conclusion, this prospective study demonstrated that slow endotracheal tube advancement during fiberoptic-guided tracheal intubation reduced the severity of postoperative sore throat. During fiberoptic-guided tracheal intubation, the edge of the distal tube tip may collide with the airway tissue and cause injury with symptoms, including postoperative sore throat, because tube advancement over the bronchoscope is blind. Based on the results of this study, we recommend a slow speed of tube advancement as a clinically simple method to reduce the severity of postoperative sore throat when performing fiberoptic-guided tracheal intubation.

## Data Availability

The datasets used and analyzed during the current study available from the corresponding author upon reasonable request.

## References

[1] Scuderi PE (2010). Postoperative sore throat: More answers than questions. Anesth. Analg..

[2] Asai T, Shingu K (2004). Difficulty in advancing a tracheal tube over a fibreoptic bronchoscope: Incidence, causes and solutions. Br. J. Anaesth..

[3] Katsnelson T, Frost EA, Farcon E, Goldiner PL (1992). When the endotracheal tube will not pass over the flexible fiberoptic bronchoscope. Anesthesiology.

[4] Tachibana N, Niiyama Y, Yamakage M (2017). Less postoperative sore throat after nasotracheal intubation using a fiberoptic bronchoscope than using a Macintosh laryngoscope: A double-blind, randomized, controlled study. J. Clin. Anesth..

[5] Cherng CH, Wong CS, Hsu CH, Ho ST (2002). Airway length in adults: Estimation of the optimal endotracheal tube length for orotracheal intubation. J. Clin. Anesth..

[6] Pak HJ, Hong BH, Lee WH (2010). Assessment of airway length of Korean adults and children for otolaryngology and ophthalmic surgery using a fiberoptic bronchoscope. Korean J. Anesthesiol..

[7] Seo JH (2013). Comparison of techniques for double-lumen endobronchial intubation: 90° or 180° rotation during advancement through the glottis. Br. J. Anaesth..

[8] Stout DM, Bishop MJ, Dwersteg JF, Cullen BF (1987). Correlation of endotracheal tube size with sore throat and hoarseness following general anesthesia. Anesthesiology.

[9] Tazeh-Kand NF, Eslami B, Mohammadian K (2010). Inhaled fluticasone propionate reduces postoperative sore throat, cough, and hoarseness. Anesth. Analg..

[10] Koga K, Asai T, Latto IP, Vaughan RS (1997). Effect of the size of a tracheal tube and the efficacy of the use of the laryngeal mask for fibrescope-aided tracheal intubation. Anaesthesia.

[11] Jones HE, Pearce AC, Moore P (1993). Fibreoptic intubation. Influence of tracheal tube tip design. Anaesthesia.

[12] Kristensen MS (2003). The Parker Flex-Tip tube versus a standard tube for fiberoptic orotracheal intubation: A randomized double-blind study. Anesthesiology.

[13] McHardy FE, Chung F (1999). Postoperative sore throat: Cause, prevention and treatment. Anaesthesia.

[14] Turkstra TP, Smitheram AK, Alabdulhadi O, Youssef H, Jones PM (2011). The Flex-Tip™ tracheal tube does not reduce the incidence of postoperative sore throat: A randomized controlled trial. Can. J. Anaesth..

[15] Jense HG, Dubin SA, Silverstein PI, O'Leary-Escolas U (1991). Effect of obesity on safe duration of apnea in anesthetized humans. Anesth. Analg..

[16] Arthurs G, Sudhakar M (2005). Carbon dioxide transport. Contin. Educ. Anaesth. Crit. Care Pain.

[17] Dang D, Robinson PC, Winnicki S, Jersmann HP (2012). The safety of flexible fibre-optic bronchoscopy and proceduralist-administered sedation: A tertiary referral centre experience. Intern. Med. J..

